# Seasonal and age-dependent differences in mercury concentrations in *Apodemus* sp. in the north-western region of Slovakia

**DOI:** 10.1007/s11356-023-31802-z

**Published:** 2024-01-10

**Authors:** Lenka Zábojníková

**Affiliations:** Institute of High Mountain Biology, University of Žilina, Tatranská Javorina 7, 059 56 Tatranská Javorina, Slovakia

**Keywords:** Mercury, Contamination, *Apodemus* sp., Seasonality, Hair, Blood, Internal organs

## Abstract

Pollution of ecosystems by heavy metals such as mercury is currently a great concern. Mercury (Hg) can be released into the environment anthropogenically, but it is also naturally present in small quantities in all environmental compartments. Many different factors contribute to different rates of Hg deposition in animal bodies. The aim of this work is to describe how Hg concentrations in the bodies of small rodents change throughout the season at a site where massive anthropogenic pollution is not expected. Mice of the genus *Apodemus* were sampled during the whole year. Samples of blood, hair, liver, kidney, and brain were analyzed. Total Hg concentrations were measured by DMA-80. The mean Hg concentrations in examined organs were in the order hairs > kidney > liver > blood > brain, and their values decreased from 0.0500 to 0.0046 mg kg^−1^ dry weight. Males and females did not differ in contamination levels, but age-dependent differences in Hg concentrations were found. It was also identified how Hg concentrations in different organs correlate with each other. Different levels of seasonal variability were detected in Hg concentrations in blood, hair, and kidney.

## Introduction

Mercury (Hg) occurs naturally in the environment. It is released into the atmosphere during volcanic activity and to a lesser extent through geothermal springs (Jitaru and Adams 2004). Forest fires and soil erosion, often increased anthropogenically, are also responsible for release of mercury into the atmosphere or aquatic ecosystems. Another artificial sources of mercury pollution are fossil fuel combustion, gold mining, non-ferrous metallurgy, cement production, waste incineration, and caustic soda production (Pirrone et al. 2010). Measures to reduce greenhouse gases appear to be effective strategies to reduce mercury emissions, as coal combustion for energy purposes is a significant source of considerable amount of anthropogenic emissions, both CO_2_ and Hg (Rafaj et al. 2013). Mercury is present in the atmosphe mainly in its elemental form. It can be transported over long distance and oxidized to a form that is deposited to ecosystems (Selin 2009).

Mercury can be present in animal bodies, but no biological function is known and all forms are highly toxic for animal organisms (Gochfeld 2003). The organic form methylmercury (CH_3_Hg^+^) is especially dangerous; it can be in a form of monomethylmercury (MMeHg, MeHg) or dimethylmercury (DiMeHg, Me_2_Hg). The toxicity of heavy metals generally lies in the replacement of calcium in the macromolecules of structural proteins. Divalent ionic form Hg^2+^ and methylmercury show strong adhesion to the thiol group (–SH), present for example in the amino acids of brain cells — neurons and glial cells (Bjørklund et al. 2017). If the –SG group is occupied by some form of mercury, it can cause disruption or blockage of the function of membrane and tubular systems. The affinity for the –SH group is also manifested in proteosynthesis. Mercury is deposited in the smooth endoplasmic reticulum, damaging the granular ER and causing secondary changes in the structure of DNA and RNA, and thus in the structure of the ribosomes themselves (Bjørklund et al. 2017). The ionic form has a good accumulation in the kidney (Berndt et al. 1985). MeHg persists longer in the body than ionic forms because the covalent bond between the mercury atom and the methyl group is very strong (Jitaru and Adams 2004). The liver is thought to be the main organ where demethylation of MeHg takes place (Yasutake and Hirayama 2001; Khan and Wang 2010; Manceau et al. 2021). Demethylation also occurs in glial cells of the nervous system. This may explain the delay in neurological symptoms of methylmercury poisoning (Syversen and Kaur 2012). Produced Hg^2+^ is the cause of significant oxidative stress. In addition, Hg^2+^ disrupts calcium and glutamate homeostasis (Bjørklund et al. 2017). During hair growth, Hg binds to –SH groups present in the amino acids that make up keratin (Clarkson et al. 2007). This mechanism effectively aids detoxification of the body.

The great danger of methylmercury lies especially in its persistence in the body, and it tends to accumulate in the body rather than being excreted. This is manifested by accumulation in the food chain (Gochfeld 2003). This biomagnification is observed in aquatic environments as well as in terrestrial ecosystems. In general, higher-order consumers show higher concentrations of mercury in the organs than lower-order consumers. Aquatic top predators are particularly at risk, where the concentration level can reach up to 10^6^ times greater (Leopold et al. 2010) compared to the concentration of mercury in the ambient environment.

Rodents, which belong to small terrestrial mammals, are an important link in the food chain (Gerstenberger et al. 2006) and a trophic base for predators. These animals can also accumulate toxins in their bodies, but do not usually reach such high concentrations as, for example, otters (Mierle et al. 2000) or marine mammals (Cardellicchio et al. 2002), and are often used as bioindicators of overall pollution. Good bioindicators of pollution are organisms with restricted home range and low migration rate, because their body condition reflects the conditions in their home range (Lord et al. 2002; Zarrintab and Mirzaei 2017). The indicator should be widespread so that regional differences can also be examined using the same model organism. The indicator should also be relatively resistant to the effects of the contaminant, because if it shows symptoms of disease even at low concentrations, its viability is reduced, causing difficulties in sampling.

There are many published studies on mercury concentrations in organs. A common focus of research is comparing sites, especially in terms of habitat (e.g., Komov et al. 2017; Peterson et al. 2021) or anthropogenic pollution (e.g., Sánchez-Chardi et al. 2007a, b; Durkalec et al. 2015). Due to their ability to accumulate contaminants in their fur, some mammals have been used as indicators for environmental monitoring. For this purpose, hair is a suitable material used to determine or estimate the concentration of the contaminant in other organs whose collection would require euthanasia. Hair analysis has the advantage of being primarily non-invasive (Gerstenberger et al. 2006), but the concentration in hair does not always correlate with concentrations in other organs such as liver (Lord et al. 2002). Especially in the case of an excessive contaminant load in the organism, the hair analysis method is no longer sufficient — it is only sufficient to identify the most critical sites and at this place sampling by an invasive method should be performed. Blood is easy to take and does not need to be collected lethally, but it has been shown that blood is not such a good indicator of long-term contamination, because it reflects a short-term contaminant status (Yates et al. 2014), influenced mainly by recent food intake. Blood samples are very useful when comparing seasonal changes, when it comes to seasonal food availability and variability in environmental conditions.

In most of the studies already published, samples were taken once, and less is known about changes over the course of the season that may disrupt the relationship between Hg concentrations in organ and hair. It is therefore questionable to what extent hair Hg concentration is really correlated with concentrations in the organs, taking into account seasonal moult and other factors in particular.

As a model organism, two abundant and easily trapped mouse species *Apodemus sylvaticus* and *Apodemus flavicollis*, living in a natural environment, were selected. Both species inhabit the same or very similar types of habitats and have similar morphology and food preferences (Abt and Bock 1998), so they were included together as one group. They were chosen mainly because they are easily trapped even in larger quantities throughout the season and because of their small size. Small mammals have a relatively large body surface relative to weight, so a large percentage of the weight is fur. In laboratory mouse, hair together with skin makes up about 14% of the total body weight (Barnett 1965). Therefore, it is assumed that more contaminants will be displaced into more mass of hair. Also, their short lifespan and rapid metabolism eliminate the effects of age and long-term storage of the contaminant in the body.

The aim of the research was to detect how mercury concentrations vary in the bodies of mice in the five types of organs analyzed (blood, hair, liver, brain, kidney) depending on tissue, sex, age, and season, also to see how concentrations of Hg in the organs correlate with each other and with morphological parameters.

## Material and methods

### Site characteristics

Animal samples were collected in the north-western region of Slovakia at a site in the cadastral area of the municipalities of Považská Bystrica, part Považská Teplá, and Plevník-Drienové (N49.15365° E18.47638°, altitude range 314–425 m a.s.l.). The area is managed by the community forest association, where small-scale forestry activities take place. The vegetation cover of the area consists mainly of planted trees with a predominance of beech *Fagus sylvatica* and spruce *Picea abies*, with a smaller representation of other species. The extensive character of timber logging contributes to the formation of mosaic habitats of small areas of forests and groves in different stages of succession, ecotones with bushes of rose *Rosa canina*, hawthorn *Crataegus* sp., black elder *Sambucus nigra*, blackthorn *Prunus spinosa*, common dogwood *Cornus sanguinea*, and blackberry *Rubus* sp. Landscape character is completed by regularly mown hay meadows, fields of agricultural crops, and private orchards.

This site was chosen mainly for practical reasons. Good accessibity and knowledge of the terrain allowed regular and routine trapping every month and collection of samples in sufficient quantities. Another reason was the low expected pollution. There is no artificial point source of pollution on the site and in the immediate vicinity that have been shown to have an impact, such as the landfill or the remains of mining activities. The amount of mercury emissions release into the atmosphere in Slovakia has been below 600 kg per year since 2009 (Jonáček et al. 2023). The territory can potentially be affected by transboundary long-distance transport of pollutants from industrial areas in the Czech Republic and Poland (Maňkovská et al. 2017).

### Sampling

Sampling began on 4th December 2020 and continued throughout the following year, ending on 26th January 2022. Sample collection was performed using Sherman traps. The traps were set up in irregular lines, depending on the assumption of mouse presence and passability of the terrain, at a distance of approximately 3–4 m. Trap locations were selected with respect to terrain availability and expected abundance of mice. Preference was given to places, and microhabitats with shrubbery vegetation were preferred, as well as areas in the early stages of secondary succession, i.e., clearings with blackberry *Rubus fruticosus*, and also places with the occurrence of naturally uprooted trunks and stumps, and plantations of spruce saplings. Remote locations away from busy sidewalks, visited by people and motor vehicles, showed a higher capture success rate. In special cases, traps were placed closer to each other when a successful capture was anticipated, such as in case of the exit of the burrows close to each other, which indicate high abundance of species of interest.

As a bait and source of food, pieces of apple or watermelon peel, or occasionaly other vegetables/fruits, or pieces of spruce or pine cones with seeds were used, depending on seasonal availability. During the winter months, pieces (approximately 5 × 5 cm) of partially preserved sheep fur were placed into the traps to provide thermal insulation and prevent hypothermia. Traps were checked early in the morning as soon as possible. Optimally, 30 or more mouse samples were collected every month.

After captured, live animals were transferred from the trap to a plastic bag and then anesthetized with isoflurane applied to cotton wool. Blood was taken using a micro hematocrit capillary (75 µl) by the retro-orbital method while narcotized. After the bleeding was stopped, a sample of hair was taken by cutting off from an area of approximately 0.5 cm^2^ of skin, from the dorsal-cranial region. Finally, sex, age, and morphometric data — weight and body length — were obtained. Age was determined visually during the handling with an animal, by size, sexual activity, and coat. Small size indicated a juvenile individual. Additionally, the fur of immatures is less distinctive in colour and softer to the touch (observed in previous trapping experiences). During the breeding season, visible signs of sexual activity during the breeding season were also helpful in determining age, indicating an adult (enlarged testes in males, visible nipples in females). Weight of animals was measured by weighing scale (100 g pesola) with the accuracy of 1 g. The animal placed in the plastic bag was weighed together with the bag; after release, the weight of empty bag (together with possible dirt such as faeces and bait remains) was deducted. Body length (from rostrum to anus) was measured during anesthesia, using a linear ruler with the accuracy of 1 mm. In some cases, measurement was not able to perform when the animal was too much active. After all the procedures had been carried out, the animal was released at the place where it was captured.

It was originally intended to collect only samples of blood and hair and release the animals after sampling. Despite the efforts to minimalize animal mortality, some animals were found dead in the traps or died accidentally during blood collection. No animal was intentionally killed. These dead individuals were used for organ analysis. The carcasses were stored in a freezing box at − 20 °C. In most cases, it was still possible to take a blood sample if the animal had not been dead for a long time.

A total of 373 individuals, including re-traps, belonging to species yellow-necked mouse *Apodemus flavicollis* (Melchior, 1834) or wood mouse *A. sylvaticus* (Linnaeus, 1758), were trapped. As these species belong to the same genus and are often difficult to distinguish in the field, the determination into the species was omitted and left only at the genus level as *Apodemus* sp. All individuals were sampled for hair, except in the case of three adult individuals that were clearly identified as re-traps (clipped hair).

### Sample storage and preparation

Right after blood collection, a drop of fresh blood was allowed to dry at room temperature to obtain a dry sample. Blood was dried on a clean Petri dish or microscope slide. Hair samples were left to dry out, if necessary, and then stored in zipped plastic bags. Before analysis, hair and blood samples were stored protected from dust and other impurities. Hair samples were not washed and were analyzed without any pre-treatment. The carcasses of dead animals were dissected in the laboratory to obtain a sample of liver, both kidneys, and brain, if the condition of the carcass allowed. The dissected organs were rinsed by distilled water to remove residual blood, hair, and other possible impurities. Subsequently, the samples of internal organs were dried in an IF160Plus laboratory Incubator (Memmert, Germany) at 50 °C for 24 h (FAN 20%).

### Laboratory analysis

A KERN 770 balance (KERN, Germany) was used to determine the weight of the samples with an accuracy of 0.0001 g. The weight of the sample material on a dry basis was in the range of 0.0010 g and not more than 0.0200 g, depending on tissue density and expected concentration. The concentration of total Hg (THg) was detected using the two-cell analyzer DMA-80 (Milestone, Italy) with nickel boats. The temperature settings were as follows: 650 °C for combustion, 615 °C for the catalyst, and 125 °C for the cuvette. NCS ZC 7001 beef liver (CHNACIS, China) was used as a reference material to ensure the accuracy of the measurement. The cleaning of the nickel boats was done by performing a blank boat analysis. After every second use of the boat, a blank boat analysis was performed. Occasionally, empty runs were performed twice or more frequently after processing a high mercury sample, which allowed removal of residual mercury that might otherwise affect results in a subsequent sample.

### Statistical analysis

The normal distribution of all data groups compared was tested by the Shapiro-Wilk test. Since the data did not have a normal distribution, nonparametric tests were used for group comparisons. Comparisons between males and females and between age classes (adults and subadults) were made by the Mann-Whitney *U* test for comparison between two independent groups. Differences between concentrations in organs and the effect of seasonality were tested by the Kruskal-Wallis test. Significantly different couples were detected by post-hoc multiple comparison of mean ranks. It was also investigated how THg concentrations in organs correlate with each other and with morphometric variables. The strength and significance of correlations were detected. Correlations and differences between groups were accepted as statistically significant at *p* ≤ 0.05.

The Shapiro-Wilk test was performed in PAST 4.03 (Hammer et al. 2001); other analyses (Mann-Whitney *U* test, Kruskal-Wallis test, and multiple comparison of mean samples, correlations) were performed by Statistica Release 7.0 (StatSoft Inc. 2008).

## Results

### Mercury concentrations in organs

Descriptive statistics of THg concentrations in mouse organs are shown in Table 1. The mean THg concentrations in the organs were in the order hair > kidney > liver > blood > brain. There were found differences among five types of organs analyzed. Significantly different THg concentrations were in couples: blood/hair; blood/liver; blood/kidney; hair/liver; hair/brain; liver/brain; liver/kidney; brain/kidney (*p* < 0.0001 all couples) (multiple comparison of mean ranks).

**Table 1:** Descriptive statistics of measured morphometric parameters and total Hg concentrations in different organs of *Apodemus* mice

	Mean ± SD	Min	Max	*n*
Weight (g)	29.0059 ± 7.1050	11.0000	61.0000	338
Body length (mm)	98.6651 ± 10.4312	59.0000	124.0000	209
Blood Hg (mg kg^−1^)	0.0065 ± 0.0083	0.0011	0.0704	307
Hair Hg (mg kg^−1^)	0.0500 ± 0.04921	0.0041	0.4831	370
Liver Hg (mg kg^−1^)	0.0101 ± 0.0074	0.0022	0.0548	162
Brain Hg (mg kg^−1^)	0.0046 ± 0.0031	0.0010	0.0171	159
Kidney Hg (mg kg^−1^)	0.0337 ± 0.0295	0.0062	0.1668	162

### Effect of sex

Of all 373 individuals of *Apodemus* mice, 183 were identified as female, 177 individuals were male, and sex of 13 animals was not determined. The effect of sex on weight, body length, and THg concentrations in organs was tested (Table 2). A significant difference was detected between males and females when comparing morphometric parameters. Males were significantly larger and heavier than females (Mann-Whitney *U* test). There was no significant difference between the sexes when comparing THg concentrations in any of the five organs examined (Mann-Whitney *U* test).

**Table 2:** Descriptive statistics (mean ± SD) and comparison of morphological parameters and organ THg concentrations between males and females of *Apodemus* mice

	Males	*n*	Females	*n*	*p*
Weight (g)	31.7812 ± 6.5796	160	26.4583 ± 6.5960	168	< 0.0001
Body length (mm)	102.6571 ± 9.3663	105	94.7228 ± 9.8612	101	< 0.0001
Blood Hg (mg kg^−1^)	0.0065 ± 0.0099	150	0.0065 ± 0.0067	147	NS
Hair Hg (mg kg^−1^)	0.0482 ± 0.0495	176	0.0530 ± 0.0502	181	NS
Liver Hg (mg kg^−1^)	0.0099 ± 0.0083	72	0.0102 ± 0.0067	87	NS
Brain Hg (mg kg^−1^)	0.0045 ± 0.0032	70	0.0048 ± 0.0032	85	NS
Kidney Hg (mg kg^−1^)	0.0311 ± 0.0297	72	0.0363 ± 0.0297	87	NS

NS refers to non-significant (*p* > 0.05) difference between groups (Mann–Whitney *U* test)

### Effect of age

Of all 373 individuals, 343 were adults and 30 individuals were immatures. A significant difference between adult and immature mice was demonstrated when comparing THg concentrations in blood, hair, liver, and brain (Table 3). Adults had significantly more THg in the hair, whereas immature individuals had higher THg concentrations in the mentioned soft tissues (Mann-Whitney *U* test).

**Table 3:** Descriptive statistics (mean ± SD) and comparison of organ THg concentrations between immatures and adults of *Apodemus* mice

	Immatures	*n*	Adults	*n*	*p*
Blood Hg (mg kg^−1^)	0.0067 ± 0.0039	23	0.0065 ± 0.0086	284	0.0466
Hair Hg (mg kg^−1^)	0.0392 ± 0.0696	30	0.0510 ± 0.0470	340	< 0.0001
Liver Hg (mg kg^−1^)	0.0116 ± 0.0029	14	0.0100 ± 0.0077	148	0.0171
Brain Hg (mg kg^−1^)	0.0059 ± 0.0032	12	0.0045 ± 0.0031	147	0.0319
Kidney Hg (mg kg^−1^)	0.0300 ± 0.0164	14	0.0340 ± 0.0305	148	NS

NS refers to non-significant (*p* > 0.05) difference between groups (Mann–Whitney *U* test)

### Correlations

Correlations between weight, body length, and THg concentrations in organs were examined. Correlations of THg concentrations with morphometric data did not prove to be significant, but the correlation between weight and body length and five correlations between THg concentrations in organs with each other were detected. The correlation coefficient and level of significance are presented in Table 4. All individuals including adults and immatures were used.

**Table 4:** Correlations between variables (morphometric parameters and THg concentrations in organs) of mice (*Apodemus* sp.)

	Body length (mm)	Blood THg (mg kg^−1^ dw)	Hair THg (mg kg^−1^ dw)	Liver THg (mg kg^−1^ dw)	Brain THg (mg kg^−1^ dw)	Kidney THg (mg kg^−1^ dw)
Weight (g)	0.8079 < 0.0001	− 0.0145 NS	− 0.0436 NS	− 0.0712 NS	− 0.1660 NS	− 0.0297 NS
Body length (mm)		− 0.0388 NS	0.0016 NS	− 0.2022 NS	− 0.2615 NS	− 0.0162 NS
Blood THg (mg kg^−1^ dw)			0.0395 NS	0.1483 NS	0.0557 NS	0.1021 NS
Hair THg (mg kg^−1^ dw)				0.1804 0.0229	0.1184 NS	0.2019 0.0107
Liver THg (mg kg^−1^ dw)					0.3566 < 0.0001	0.7498 < 0.0001
Brain THg (mg kg^−1^ dw)						0.2133 0.0073

Correlations are shown as *r*-values (upper) and *p*-values (below, if significant). NS refers to non-significant (*p* > 0.05) correlation

### Seasonality

**Fig. 1:**
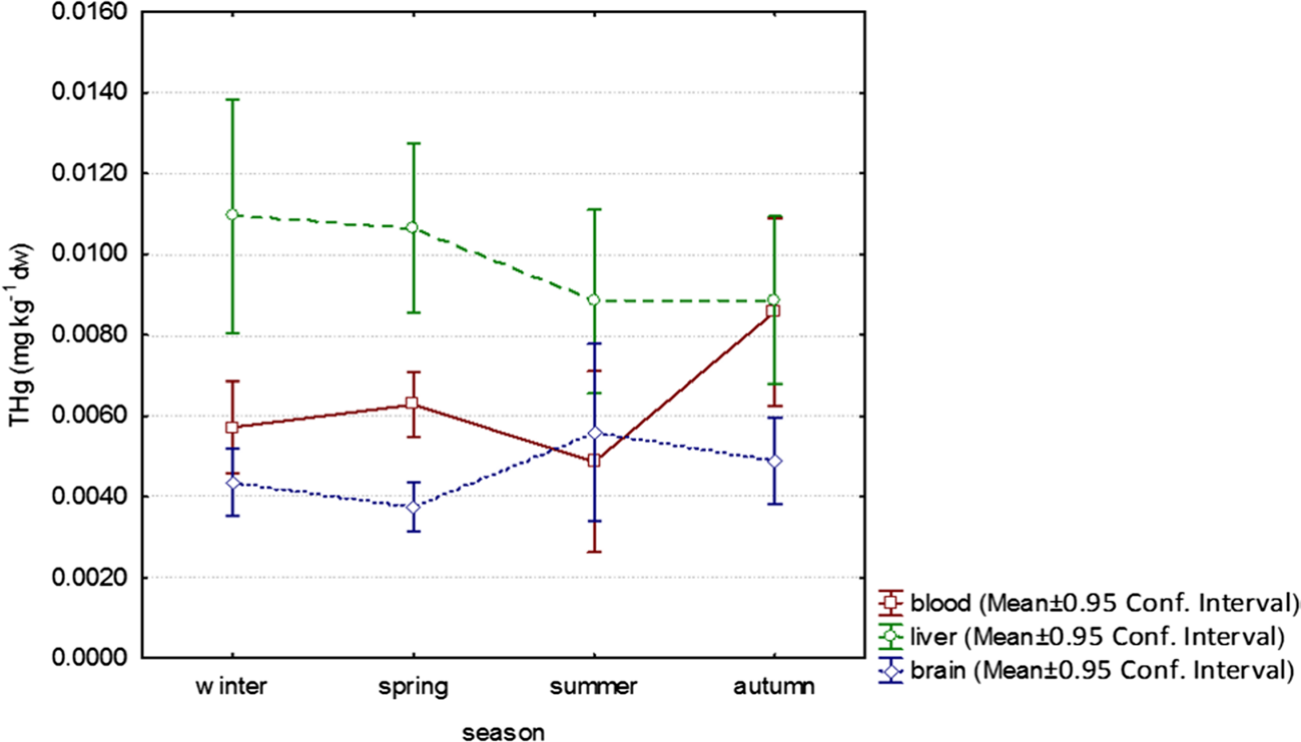
Total Hg concentrations (mg kg^−1^ dw) in blood, liver, and brain of mice (*Apodemus* sp.) in relation to season

**Fig. 2:**
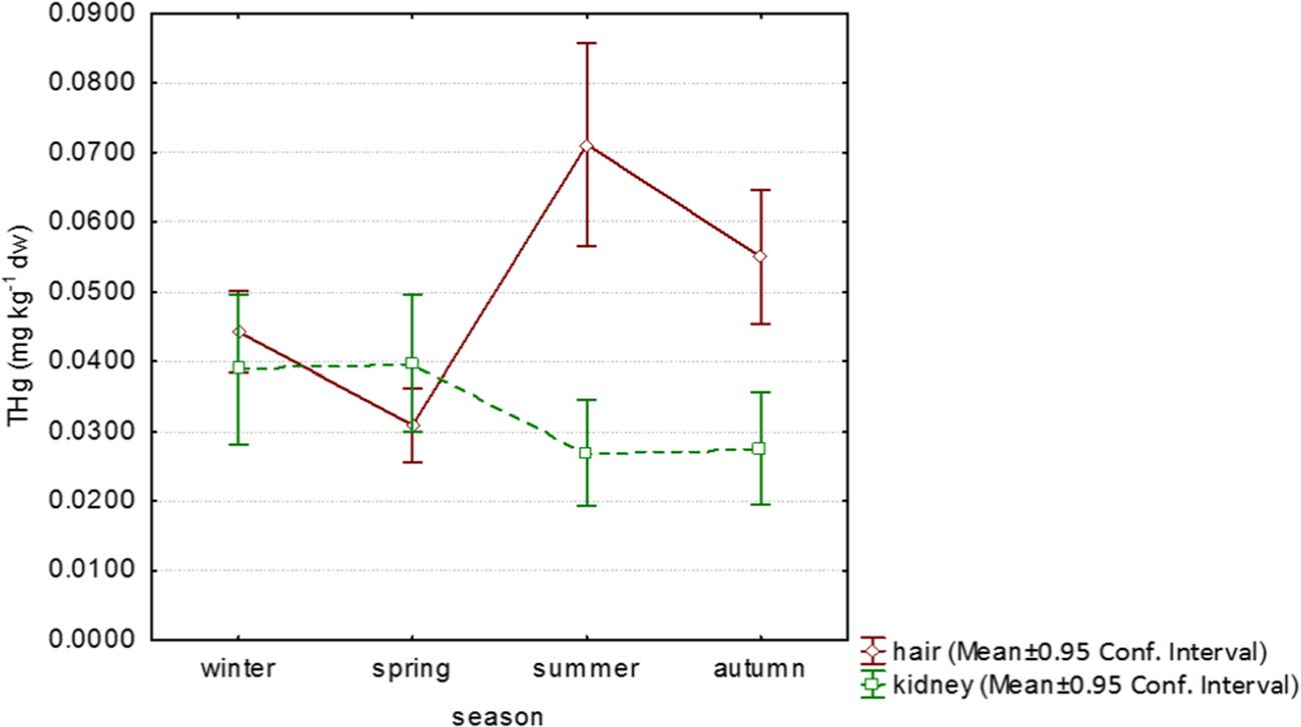
Total Hg concentrations (mg kg^−1^ dw) in hair and kidney of mice (*Apodemus* sp.) in relation to season

To compare the effect of seasonality, only individuals identified as adults were used. Four seasonal categories were created. Figure 1 shows seasonal changes in THg concentrations in blood, liver, and brain. Figure 2 shows the changes in THg concentration over the season in hair and kidney.

Significantly different THg concentrations (mg kg^−1^) in blood were in couples: winter (0.0057 ± 0.0046, *n* = 66)/summer (0.0049 ± 0.0094, *n* = 84) (*p* = 0.0009); spring (0.0066 ± 0.0028, *n* = 62)/summer (*p* < 0.0001); summer/autumn (0.0085 ± 0.0110, *n* = 95) (*p* < 0.0001) (multiple comparison of mean ranks).

Detected significant differences in THg concentrations (mg kg^−1^) in hair were winter (0.0443 ± 0.0287, *n* = 92)/spring (0.0294 ± 0.0250, *n* = 90) (*p* = 0.0038); winter/summer (0.0680 ± 0.0659, *n* = 91) (*p* = 0.0243); spring/summer (*p* < 0.0001); and spring/autumn (0.0580 ± 0.0555, *n* = 97) (*p* < 0.0001) (multiple comparison of mean ranks).

THg concentrations (mg kg^−1^) in kidney differed significantly in a couple: spring (0.0377 ± 0.0266, *n* = 46)/autumn (0.0276 ± 0.0277, *n* = 74) (*p* = 0.0368) (multiple comparison of mean ranks).

There were no significant seasonal differences in THg concentrations in the liver and brain (Kruskal–Wallis test).

## Discussion

### Mercury concentrations in organs

Mercury concentrations in all organs were generally low. According to Sánchez-Chardi et al. (2007a), a concentration of 30 mg kg^−1^ of mercury in the liver and kidney is considered to be the threshold value for mammalian intoxication. Even a value higher than 1.1 mg kg^−1^ is an indicator of an environmental problem for wild mammals. All values obtained were below these limits. This is due to the site, which cannot be considered as highly polluted, and also to the low trophic level of the species studied small rodents, which are the food base for higher-order consumers, which are also an intermediate step for the transfer of contaminants to higher levels of the trophic chain (Gerstenberger et al. 2006). *A. flavicollis* and *A. sylvaticus* are omnivores with a predominantly plant-based food (Hansson 1971), and therefore contain lower concentrations of mercury in the organs than mesopredators (Peterson et al. 2021) or top predators (Dainowski et al. 2015, Treu et al. 2017, Kalisinska et al. 2021).

Table 5 shows examples of mercury concentrations detected in organs of *A. flavicollis* and *A. sylvaticus* in other regions.

**Table 5:** Examples of mercury concentrations measured in other regions in mice *Apodemus sylvaticus* and *A. flavicollis*

Species	Species name	Region, country	Habitat/contamination	Year, season	Organ	Concentration	Reference
Wood mouse	*Apodemus sylvaticus*	Not specified	Near chlor-alkali works	1974, spring/summer	Hair	0.7800 ± 0.1200	Bull et al. 1977
					Liver (ww)	0.2300 ± 0.0700	Bull et al. 1977
					Brain (ww)	0.5500 ± 0.2800	Bull et al. 1977
					Kidney (ww)	0.5200 ± 0.1600	Bull et al. 1977
					Muscle (ww)	0.9800 ± 0.7300	Bull et al. 1977
			Control area, unpolluted	1974, spring/summer	Hair	0.1200 ± 0.0100	Bull et al. 1977
					Liver (ww)	0.0400 ± 0.0200	Bull et al. 1977
					Brain (ww)	0.0600 ± 0.0100	Bull et al. 1977
					Kidney (ww)	0.1200 ± 0.0200	Bull et al. 1977
					Muscle (ww)	0.0700 ± 0.0100	Bull et al. 1977
Wood mouse	*Apodemus sylvaticus*	Not specified	Hg and organochlorine treated wheat field	1973, October	Whole body without liver, kidney and testes (ww)	0.3900 ± 0.0400	Jefferies and French 1976
				1973, November–December	Whole body without liver, kidney and testes (ww)	0.8300 ± 0.0440	Jefferies and French 1976
Yellow-necked mouse	*Apodemus flavicollis*	Žerjav, Slovenia	Meadow, near lead smelter	2012, summer	Liver (ww)	0.3300 ± 0.2400*	Al Sayegh Petkovšek et al. 2014
		Veliki vrch, Slovenia	Mixed forest, near thermal power plant	2012, summer	Liver (ww)	0.1400 ± 0.1700*	Al Sayegh Petkovšek et al. 2014
		Črnova, Slovenia	Farmland/mixed forest, main road	2012, summer	Liver (ww)	0.0200 ± 0.0200*	Al Sayegh Petkovšek et al. 2014
		Polanc, Slovenia	Mixed forest, unpolluted	2012, summer	Liver (ww)	0.0600 ± 0.0400*	Al Sayegh Petkovšek et al. 2014

Concentrations are shown as mean ± standard error (* refers to mean ± standard deviation). All concentration values were converted to mg kg^−1^. ww = wet weight, respectively fresh weight

Among the organs studied, the highest values were measured in hair, followed by kidneys, liver, and blood, and the lowest THg concentrations were found in the brain.

Both forms of mercury are present in blood. Inorganic forms bind to blood plasma proteins, such as albumin, or form conjugates with non-protein metabolites that also contain –SH groups. This complex is transported more rapidly to target cells. The binding of Hg to blood plasma proteins is reversible (Ajsuvakova et al. 2020); therefore, the Hg concentration in blood is unstable and influenced by recent dietary uptake (Yates et al. 2014). Methylmercury tends to bind to red blood cells (Neathery and Miller 1975). Up to 95–96% of the total Hg in blood cells is in the form of methylmercury (Airey 1983).

Mercury concentrations in hair were the highest among all organs analyzed. The same was observed in minks *Neovison vison* (Evans et al. 2016), otters *Lontra canadensis* (Mierle et al. 2000), raccoons *Procyon lotor* (Lord et al. 2002), and red fox *Vulpes vulpes* (Dainowski et al. 2015), hair having higher mercury concentrations than soft tissues. Keratin, contained in mammalian hair, is a protein rich in sulfhydryl groups (McLean et al. 2009; Ye et al. 2016), to which Hg binds well (Ajsuvakova et al. 2020). Therefore, during the period of hair formation, Hg in blood is sequestered into newly forming hair (Clarkson et al. 2007). It is assumed that high abundance of –SH groups in hairs is the reason for the high accumulation of Hg in hair tissue and the resulting concentration in hairs exceeds that in blood. Incorporation of Hg, especially in the form of MeHg, into mammal hair is irreversible (Ye et al. 2016). This incorporation and later shedding of hair is a significant route of elimination of Hg from the body (Farris et al. 1993; Ye et al. 2016).

Liver and kidney are frequently used organs in ecotoxicological studies. The predominance of renal rather than hepatic THg concentration is also reported in wood mouse and bank vole *Myodes glareolus* (Bull et al. 1977, Komov et al. 2017; Ecke et al. 2020), deer mouse *Peromyscus maniculatus* (Vucetich et al. 2001), common shrew *Sorex araneus* (Komov et al. 2017), roe deer *Capreolus capreolus* and wild boar *Sus scrofa* (Durkalec et al. 2015), European mole *Talpa europaea*, water vole *Arvicola terrestris*, and muskrat *Ondatra zibethicus* (Antonova et al. 2017). However, higher renal Hg concentration may not be a strict rule. While renal Hg predominates in herbivorous and omnivorous mammals, higher liver concentrations have been reported in predators preying on aquatic organisms (Antonova et al. 2017, Treu et al. 2018, Kalisinska et al. 2021). This is mainly due to the difference in the form and origin of deposited mercury. The kidney is considered to be the target organ in the accumulation of inorganic Hg (Berndt et al. 1985), whereas organic MeHg, commonly found in aquatic ecosystems, is deposited in the liver. Therefore, animals preying on aquatic organisms have elevated levels of Hg in the liver.

Mercury in the brain can also be in both forms, ionic and methylmercury. Methylmercury can cross the blood–brain barrier, whereas ions are not capable of this transport. Therefore, the percentage of inorganic Hg in the brain is low (Friberg and Mottet 1989). Ion forms can occur in the brain as a result of oxidation of elemental form to divalent ion or demethylation of methylmercury (Bjørklund et al. 2017). Despite neurotoxic effects (Chang 1977; Ajsuvakova et al. 2020), concentrations in the brain are relatively low compared with organs such as liver (Mierle et al. 2000).

### Effect of sex

There was a significant difference between the weights of male and female mice. Males were on average larger than females. It is reported that ecological differences such as home range size (Ecke et al. 2020) and food preferences (Lurz et al. 2017; Chételat et al. 2020) can contribute to differences in mercury accumulation and excretion between sexes. Factors such as maternal transfer during gravidity and lactation (Durkalec et al. 2019; Chételat et al. 2020) and higher capacity for demethylation (Robinson et al. 2011) can cause females having lower levels of Hg in their bodies. Not such effect was observed; sex did not significantly affect Hg concentrations in individual organs. The same was observed in bodies of bank voles and shrews (Komov et al. 2017), in hair of raccoons and striped skunks *Mephitis mephitis* (Peterson et al. 2021), muskrats (Stevens et al. 1997), and two of the three populations of red squirrels *Sciurus vulgaris* (Lurz et al. 2017), without the difference between males and females.

### Effect of age

There were significant differences between adult and immature mice indicating that immatures have more mercury in the blood, liver, and brain, and adults have more contaminated hair (Table 3).

Similarly Sánchez-Chardi et al. (2007b) reported that juveniles have more metals in their liver than adults. The explanation is that animals absorb more metals into the body at juvenile age and later in adulthood their intestinal absorption decreases, although this rule applies more to other heavy and essential metals. Growing juveniles compensate for their high energy needs by increasing their food intake, from which contaminants are deposited in the body. The opposite of this statement is that of Antonova et al. (2017), who found an increase in Hg concentrations in adult insectivores and rodents compared to juveniles. This increase in liver and skeletal muscle was more observable in insectivorous water shrew, while the increase in kidney was more pronounced in water voles and muskrats. This is explained by the fact that more persistent MeHg, originated from animal food, is more likely to be deposited in liver and muscles. Also, in many bird species, adults have more MeHg in the body (Ackerman et al. 2011). Methylmercury is a compound, not an element, so it acts differently in the body. The growth of the body does not allow the rapid accumulation of contaminants. As body mass increases, the concentration dilutes with increasing weight. After reaching adulthood, growth slows down, so dilution with increasing weight no longer works as efficiently as it does at juvenile age and the contaminant accumulates faster in a slower growing body than it can dilute (Ackerman et al. 2011). Another factor influencing the different levels in adults and juveniles may be the difference in preferred diet.

The low Hg levels in juvenile mouse hair might be caused by short duration of juvenile coat compared to persistent, exogenously enriched adult coat, or to the low initial concentration of Hg in blood during juvenile coat formation.

### Correlations

Mouse body length and weight correlated well with each other, but no significant correlations were observed between morphological parameters and THg concentrations (Table 4). Both directly proportional and inversely proportional (Gerstenberger et al. 2006; Yates et al. 2014; Durkalec et al. 2019; Peterson et al. 2018, 2021) relationships between Hg concentration and morphological parameters have been reported in different animals. However, a relationship between morphometric parameter and concentration may also be influenced by both variables.

There was a strong and significant correlation (*r* = 0.7498) between liver and kidney THg concentrations. Despite the fact that a different form of Hg is stored in the kidneys and another form is deposited in the liver (Pokorny and Ribarič-Lasnik 2002), there is the strongest linear relationship between these organs. It is stated that this is due to the active tissue metabolism of both organs and the direct connection with the bloodstream (Treu et al. 2018). In liver, demethylation of methylmercury occurs and the ionic form is then transported either by bile to the intestinal tract to be excreted in the feces or by blood to the kidneys to be excreted in the urine (Chételat et al. 2020). This connection and the evidence of strong correlation between correlations in these two organs imply a direct dependence of renal Hg concentrations on processes taking place in liver.

The blood level of Hg indicates a short-term state of the contaminant in the body (Yates et al. 2014). It reflects the amount of Hg that has not yet been deposited in organs or excreted. No strong and significant correlations were observed in mice when comparing Hg concentrations in blood and other organs. Therefore, blood does not serve as a good indicator of whole-body contamination in *Apodemus* mice.

Several studies have investigated Hg levels in hair because examination of organs of abdominal cavity can only be performed after euthanasia, whereas hair sampling is less invasive (Gerstenberger et al. 2006). The ability of hairs to predict internal body burden by contaminant (Gerstenberger et al. 2006, Treu et al. 2018) has some limitations. In most previous studies, samples were collected regardless of seasonal variations and differences. Despite different seasonal trends, hair THg concentrations correlated with THg concentrations in the liver (*r* = 0.1804) and kidney (*r* = 0.2019). The long residence time of MeHg (Grandjean and Herz 2015) deposited mostly in the liver (Kalisinska et al. 2021) may also be the reason why the Hg concentration in the liver correlated well with the Hg concentration in hair in mice. Liver methylmercury and Hg in keratin of hair tend to be similarly persistent and thus in a directly proportional linear relationship. The strong correlation between THg concentrations in the liver and kidney (*r* = 0.7498) was also evident in the relationship with hair. Therefore, hair was also correlated well with kidney (*r* = 0.2019).

Brain mercury levels correlated well with both THg concentrations in the liver (*r* = 0.3566) and kidney (*r* = 0.2133); also, hair mercury correlated with mercury in the liver and kidney, but THg concentrations in hair and brain were not significantly correlated with each other. This finding is surprising because according to Clarkson et al. (2007), “the hair follicle accumulates the same transportable species of mercury as that which enters the brain”. The results show that the form of deposited Hg (inorganic/MeHg) is not critical for the correlations. THg concentrations in organs in which the same form is deposited (brain, hair) were not correlated with each other; on the contrary, the strongest correlation was found between organs that differ in the form of accumulated Hg (liver, kidney).

### Seasonality

Seasonal changes in blood THg concentrations have been detected, showing that in summer the blood is least burdened by mercury. Hg in the blood represents a short-term state, primarily influenced by recent dietary intake (Yates et al. 2014). Wood mice and yellow-necked mice are able to adapt to different diets depending on seasonal availability. In the summer period, their main diet consists of larval and adult insects (Montgomery and Montgomery 1990); in the later autumn period, when vegetation produces seeds, they switch to seed food (Hansson 1971). Proteins derived from animal food appear to be involved in increased Hg excretion and thus aid detoxification, as has been found in laboratory mice (Adachi et al. 1992).

The seasonal course of THg concentrations in hair indicates that the Hg concentration is lower in spring and higher in summer. The current hair mercury concentration is likely shaped by three main factors: past conditions during the season of hair formation in the time of moult (Mierle et al. 2000; Yates et al. 2014), additional enrichment by mercury from the environment (Sobańska 2005), and abrasion of the most contaminated distal hair ends (Peterson et al. 2021). It is likely that exogenous deposition caused a rapid increase in hair mercury concentration in summer months.

There was a difference between THg concentrations in the kidney in the spring and autumn seasons, showing that concentrations were highest in the spring season. A contradiction to this statement is the finding by Pokorny and Ribarič-Lasnik (2002) in the roe deer, where the highest values were recorded in August–September. This is explained by higher food intake in the period of the coming autumn, but this is only true for herbivores with low seasonal variation in food intake.

The increase in Hg concentration from summer to autumn observed in blood was absent in both kidney and liver. An explanation may be that during the autumn molt, Hg was deposited in the newly emerging coat. MeHg binds to keratin during hair formation (Clarkson et al. 2007); hence, there was no enrichment in the liver and kidney.

## Conclusion

Several animal species are used as indicators for the study of contaminants such as mercury, in the environment. The amount of contaminant in their bodies reflects environmental exposure, but concentrations of contaminants in animal organs are influenced by other factors such as the physiological response of the organism and changing seasonal conditions that alter the availability of the contaminant in the environment. Two small species of mice, *Apodemus flavicollis* and *Apodemus sylvaticus*, were used to better understand these relationships. The results show that the mercury concentrations in the organs studied, blood and hair, and in the case of dead individuals also in the liver, brain, and kidney, vary according to the age of the individual and/or the time of sampling. The expected effect of sex was not observed. Blood has not been shown to be a very useful indicator of internal mercury exposure, as blood mercury status varies independently of other organs. In contrast, non-invasive hair sampling can replace sampling of internal organs such as liver and kidney. Further studies are needed to elucidate the causes of the observed seasonal changes in terms of changing environmental conditions and physiological or behavioral interactions that cause differential Hg accumulation in different organs.

## Data Availability

The datasets used and/or analyzed during the current study are available from the corresponding author on reasonable request. The author declares to cite any publicly available data on which the conclusions of the paper rely in the manuscript.
